# Alterations in cartilage quantification before and after injections of mesenchymal stem cells into osteoarthritic knees

**DOI:** 10.1038/s41598-021-93462-8

**Published:** 2021-07-05

**Authors:** Ichiro Sekiya, Hisako Katano, Mitsuru Mizuno, Hideyuki Koga, Jun Masumoto, Makoto Tomita, Nobutake Ozeki

**Affiliations:** 1 Center for Stem Cell and Regenerative Medicine, Tokyo Medical and Dental University, 1-5-45 Yushima, Bunkyo-ku, Tokyo, 113-8510 Japan; 2 Department of Joint Surgery and Sports Medicine, Graduate School of Medical and Dental Sciences, Tokyo Medical and Dental University, Tokyo, Japan; 3 Fujifilm Corporation, Tokyo, Japan; 4 School of Data Science, Graduate School of Data Science, Yokohama City University, Yokohama, Japan

**Keywords:** Stem cells, Musculoskeletal system

## Abstract

Several studies have reported improvement in knee pain following mesenchymal stem cell (MSC) injections for knee osteoarthritis (OA). We developed a novel 3D magnetic resonance imaging (MRI) analysis software program that provides “projected cartilage area ratios” for automatic detection of changes in cartilage amounts. The primary objective of this prospective interventional study was to compare alterations in the projected cartilage area ratio (thickness ≥ 1.5 mm) at the femoral posteromedial region between 30 weeks before and 30 weeks after synovial MSC injections. Secondary objectives were to assess the clinical scores and safety of MSC injections. Patients with OA who complained of knee pain underwent autologous synovial MSC injections into the knee at time 0 and again 15 weeks later. MRI examinations were performed at − 30, − 15, − 1, and 30 weeks. Patients showing < 3% decreases in the projected cartilage area ratio (thickness ≥ 1.5 mm) at the femoral the posteromedial region from − 30 weeks to − 15 weeks were excluded from the study. The Lysholm Knee Score, Knee Injury and Osteoarthritis Outcome Scale (KOOS), and Numerical Rating Scale (NRS) scores were evaluated at − 30, − 15, − 5, − 2, 0, 5, 10, 15, 20, 25, and 30 weeks. Five patients were excluded because 3D MRI analysis showed no cartilage loss at − 15 weeks. Ultimately, eight OA patients underwent MSC injections. The projected cartilage area ratio significantly decreased by 0.07 in the 30 weeks before MSC injections (*p* = 0.01), but no further decreases occurred in the 30 weeks after MSC injections. The projected cartilage area ratio at the femoral posteromedial region showed a significant difference between 30 weeks before and 30 weeks after MSC injections. The Lysholm Knee Score, KOOS, and NRS values improved significantly after the injections. MSC injection could not be ruled out as the cause of two adverse events: transient knee pain and itching in both hands. Fully automatic 3D MRI analysis showed that synovial MSC injections suppressed cartilage loss in patients with progressive OA.

Trial registration: Intraarticular injections of synovial stem cells for osteoarthritis of the knee (Number UMIN 000026732). Date of registration; June 1, 2017. https://upload.umin.ac.jp/cgi-open-bin/ctr/ctr_view.cgi?recptno=R000029967.

## Introduction

Magnetic resonance imaging (MRI) can directly evaluate the articular cartilage, making this imaging technique useful for understanding the pathological condition of osteoarthritis (OA) of the knee. We previously proposed the determination of “the projected cartilage area ratio” in the femoral cartilage by 3D MRI analysis as a way to understand the pathology of this most prevalent degenerative joint disease^1^. The projected cartilage area ratio is the ratio of the projected cartilage area to the total area of the region of interest (ROI). This ratio allows the detection of changes in the amount of cartilage in OA in a relatively short time simply by changing the cartilage thickness threshold. In this report, the cartilage area was extracted semi-automatically and required manual correction. We have overcome this limitation by improving the software so that it now allows fully automatic extraction of the cartilage area using deep neural networks, followed by application of the projected cartilage area ratio to the tibial cartilage^2^.

One promising strategy that may improve MRI findings in OA is the use of mesenchymal stem cells (MSCs). Reports on intra-articular injections of MSCs for the treatment of OA knees are increasing, and systematic reviews indicate that these MSCs can improve OA pain in many cases^3–5^. Some reports have described improved MRI results following intraarticular MSC injections; however, the MRI evaluations used in previous studies have included procedures such as Magnetic Resonance Observation of Cartilage Repair Tissue (MOCART)^6,7^, one-slice evaluations using 2D images^8,9^, and semiautomatic 3D analysis^10^, and these procedures might not be sufficiently objective or reliable.

We previously investigated the effects of intra-articular injections of synovial MSCs on a rat OA model. We found that the synovial MSCs, upon injection into the OA rat knees, maintained their viability without losing their MSC properties and inhibited OA progression by the secretion of trophic factors^11^. Some clinical reports have evaluated intra-articular injections of MSCs derived from intra-articular tissues, but those MSCs were derived only from the infrapatellar fat pad (IFP)^12^. The purpose of the current prospective interventional study was to compare the alterations in the projected cartilage area ratio (thickness ≥ 1.5 mm) at the femoral posteromedial region between 30 weeks before and 30 weeks after synovial MSC injections. Secondary objectives were to assess the clinical OA scores (Lysholm, KOOS, and NRS) after this treatment and the safety of the MSC injections. The underlying hypothesis for this study was that MSC injections would inhibit the cartilage loss that occurs in the progressive OA knee.

## Materials and methods

### Study design

This study was conducted in accordance with the Declaration of Helsinki and approved by the Bureau of Health and Welfare of the Ministry of Health, Labour, and Welfare, Japan after review by the Certified Special Committee for Regenerative Medicine at our university. The protocol was enrolled in a database of the National University Hospital Council of Japan (UMIN Clinical Trials Registry) and disclosed (Number UMIN 000026732).

### Inclusion and exclusion criteria

The inclusion criteria included patients (1) over 20 years of age, (2) complaining of knee pain and (3) diagnosed with knee OA by X-ray, (4) but whose pain had not been improved by injection of hyaluronic acid, and (5) whose condition had not been improved by 3 months of exercise therapy. The exclusion criteria included patients (1) whose projected cartilage area ratio (thickness ≥ 1.5 mm) at the femoral posteromedial region decreased by less than 3% from − 30 weeks to − 15 weeks, (2) who had a history of trauma, arthroscopy, or surgery in the knee to be injected, and (3) who had undergone knee injection or aspiration from the knee within the past 3 months.

### MRI scanning

An MRI system with 16-channel coils (Achieva 3.0TX, Philips, Amsterdam, Netherlands) was used at 3.0 T. The sagittal plane of the knee joint was acquired to obtain both a fat-suppressed spoiled gradient echo sequence image for cartilage and a proton weighted image for bone, with total scan durations of 7 min 30 s and 7 min 10 s, respectively. For both images, sagittal images were obtained at an in-plane resolution of 0.31 × 0.31 mm, a partition thickness of 0.36 mm (320 slices), and a field of view (head to tail × anterior to posterior) of 150 × 150 mm.

### 3D MRI analysis

The MRI DICOM data were analyzed using our novel software. Approximately three minutes after reading the MRI data, the cartilage and bone areas were automatically extracted and a 3D MRI for cartilage and bone was reconstructed. The cartilage area was then projected onto a flat surface according to the long axis, and the rotation axis of the femur and tibia was determined to allow horizontal rotation of the posterior condylar line of the femur and tibia. The projected cartilage area was quantified using the “projected cartilage area ratio,” which was the ratio of the projected cartilage area to the total area (= the region of interest, ROI).

For the ROI of the femoral cartilage, the software automatically drew one closed curve along the bone contour and then drew lines that split the projected femoral cartilage into four equal regions in the longitudinal and transverse directions. The two posterior regions almost completely covered the region from the anterior portion to the posterior portion of each meniscus, with the knee in a slightly flexed position. The two anterior segments covered approximately half of the lower part of the patellofemoral joint. For this study, only the posteromedial and posterolateral regions were analyzed; the anteromedial and anteromedial regions were not.

The ROI of the medial and lateral tibial cartilage was defined by two closed curves drawn automatically by the software based on the bone morphology. Our software provided cartilage thickness mapping based on the fat-suppressed spoiled gradient echo sequence images from the MRI. Areas with a cartilage thickness of more than 2.0 mm were displayed in white, areas with a cartilage thickness close to 0.0 mm were displayed in red, and areas without any cartilage were displayed in gray (which was also set as the color of the bone). Our software also provided an average cartilage thickness (mm) for each region. The “projected cartilage area ratio (thicknesses ≥ 1.5 mm)” was the ratio of the projected cartilage area with a cartilage thickness of 1.5 mm or more to the total area of the ROI^1^.

### Procedure for MSC injections

Approximately 2 weeks before harvesting of the synovial tissue, nearly 300 mL of whole blood was obtained using a closed-bag system (CELLAID, JMS Co., Ltd, Hiroshima, Japan) and autologous serum was prepared.

With the knee under local anesthesia, the synovium, along with subsynovial tissue on the femur at the suprapatellar pouch, was harvested with a pituitary rongeur under arthroscopic observation. Approximately 20 pieces of synovial tissue, weighing nearly 0.5 g, were collected and transferred to 10 mL Hank's Balanced Salt Solution (HBSS, Thermo Fisher Scientific, MA, USA).

The synovial MSCs were cultured in a cell processing facility at the authors’ institution. The synovium was digested in a solution of 5 mg Liberase MNP-S GMP (Roche Diagnostics, Mannheim, Germany) in 1 mL autologous serum and 4 mL water. After 3 h of gentle shaking, the digested cells were plated in approximately 60 dishes with a surface area of 150 cm^2^. The nucleated synovial cell numbers per synovium weight ranged between 5 × 10^6^ and 25 × 10^6^ cells per g synovial tissue. The cells were cultured for 14 days in alpha-minimum essential medium (α-MEM: Thermo Fisher Scientific) containing 10% autologous human serum and 1% antibiotic–antimycotic (Thermo Fisher Scientific).

We performed bacterial tests on the HBSS used to transport the synovium before culture and on the cell supernatant 8 and 14 days after culture. The result of bacterial testing after 14 days of culture was not available before the injection. We also performed nucleic acid amplification (NAT) tests for mycoplasma at 11 days after culture and an endotoxin test immediately before the injections.

Upon obtaining satisfactory results for these quality control measures, the synovial MSCs were treated with TrypLE (Thermo Fisher Scientific) at 37 °C for 10 min and harvested^13^. A sample containing 2.0 × 10^7^ primary synovial MSCs was then suspended in a mixture of 0.5 mL autologous serum and 4.5 mL lactated Ringer's solution (Lactec Injection, Otsuka Pharmaceutical, Tokyo, Japan) and then injected into the patient’s knee.

The remaining MSCs were aliquoted into freezing tubes (Sumitomo Bakelite, Tokyo, Japan) at 1 × 10^6^ cells in 400 μL α-MEM containing 5% DMSO (CultureSure DMSO; FUJIFILM Wako Pure Chemical Corporation), 1% antibiotic–antimycotic, and 10% autologous serum. The tubes were frozen overnight in a bio freezing vessel (BICELL, Japan Freezer, Tokyo, Japan) in a − 80 °C freezer, and then stored in a − 150 °C freezer^14^.

Approximately 4 weeks before the second MSC injection, another nearly 300 mL sample of whole blood was collected. One tube of frozen synovial MSCs was thawed using a frozen cell thawing device (ThawSTAR, Astero Bio, CA, USA), and the cell viability was determined by staining the cells with acridine orange for live cells and with propidium iodide for dead cells. The live and dead cells were counted with an automated cell counter (Luna-FL, Logos Biosystems, VA, USA)^15^. The viability ranged between 84 and 96%.

The thawed cells were cultured with 10% autologous human serum for 14 days, and 2 × 10^6^ synovial MSCs at passage 1 were suspended in a mixture of 0.5 mL autologous serum and 4.5 mL lactated Ringer's solution. This cell suspension was then used for the second MSC injection.

### Schedule

**Figure 1:**
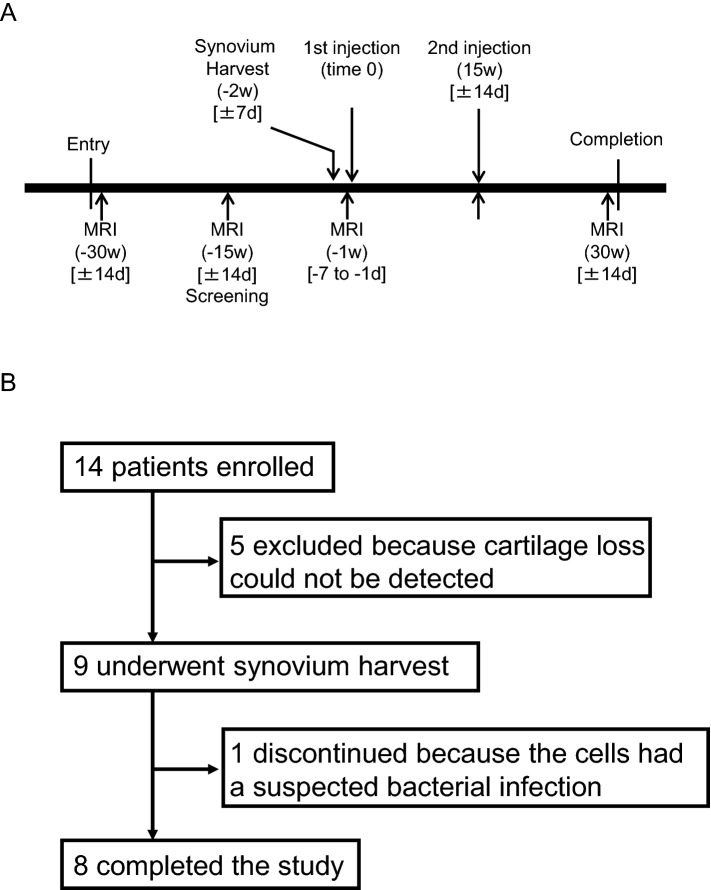
Study scheme. (**A**) Schedule for the clinical study. Square brackets indicate the allowable range. (**B**) Enrollment of patients.

Patients enrolled in the clinical study underwent MRI examinations at − 30 weeks and − 15 weeks prior to MSC injection. If their projected cartilage area ratios at the femoral posteromedial region, determined by MRI 3D analysis, had decreased by less than 3%, the patient was excluded from the study. Synovium was harvested at − 2 weeks, another MRI examination was performed at − 1 week, and the first synovial MSCs were injected one week later (time 0). The second synovial MSC injection was conducted at 15 weeks, and another MRI examination was performed at 30 weeks, when the study was concluded (Fig. 1A).

### Software for 3D MRI analysis

MRI screening between − 30 weeks and − 15 weeks was performed between April 2018 and April 2019. For those periods, cartilage extraction was corrected manually because our software was not yet sufficiently accurate^1^. Subsequently, our software accuracy improved and allowed automatic analysis of the alterations in cartilage quantification before and after MSC injections without any need for manual correction^2^.

### Evaluation of clinical scores

The Lysholm Knee Score, Knee Injury and Osteoarthritis Outcome Scale (KOOS), and Numerical Rating Scale (NRS) were evaluated at − 30, − 15, − 5, and − 2 weeks, and then again at time 0 and at 5, 10, 15, 20, 25, and 30 weeks. Analgesic use was recorded from 1 week before the clinical score evaluation. No analgesics were used for one week before the clinical score assessment.

### Assessment of adverse events

Adverse events were monitored continually at each visit. The knees were examined for redness, swelling, and changes in range of motion (ROM). Clinical laboratory values and X-ray examination were recorded at entry, at − 5 weeks, and at 30 weeks.

### Statistical analysis

Alterations in the cartilage quantifications before the injections were calculated by subtracting “the value at − 30 weeks” from “the value at − 1 week.” Alteration in cartilage quantification after the injections was calculated by subtracting “the value at − 1 week” from “the value at 30 weeks.” Because of the allowable range, the duration from the MRI at − 30 weeks to the MRI at − 1 week was 29.8 ± 0.7 weeks and the duration from the MRI at − 1 week to the MRI at 30 weeks was 28.5 ± 0.5 weeks. Alterations in cartilage quantification before and after MSC injections, as well as cartilage quantification at − 30, − 1, and 30 weeks, were compared statistically using the Wilcoxon signed rank test. Clinical scores for continuous variables between values at time 0 and at each visit were also compared using the Wilcoxon signed rank test. A P value of 0.05 was considered statistically significant. Quantitative values were presented as medians with interquartile ranges (IQR) (n = 8). GraphPad Prism8 (GraphPad Software, San Diego, CA, USA) was used for all statistical analyses.

### Ethics approval and consent to participate

This study was approved by the Certified Special Committee for Regenerative Medicine of Tokyo Medical and Dental University. Written informed consent forms were submitted by all participating patients.

### Consent for publication

A consent form will be sent upon acceptance of this paper.

## Results

### Enrollment of patients

Fourteen patients were initially enrolled in this clinical study, but five were excluded because they showed no detectable cartilage loss. Nine patients underwent synovium harvesting, but one patient was withdrawn from the study because the cells had a suspected bacterial contamination (although this bacterial infection was eventually refuted). Eight patients therefore completed the study (Fig. 1B).

### Characteristics of the patients

The eight patients included five females and three males ranging in age from 51 to 79 years and BMI from 23 to 30. The Kellgren–Lawrence radiographic OA grade was grade 2 for 4 knees, grade 3 for 3 knees, and grade 4 for 1 knee (Table 1). All patients showed medial OA.

**Table 1:** Patient demographics.

Patient	Gender	Age	Height (cm)	Weight (kg)	BMI	Injected knee	KL grade
01	F	63	160	73	29	L	3
02	M	78	169	72	25	R	3
03	F	67	146	64	30	L	3
04	M	72	171	75	26	R	2
05	F	65	159	59	23	R	2
06	F	77	154	54	23	R	2
07	F	79	162	60	23	R	4
08	M	51	162	68	26	R	2
Median		70	161	66	26		
1st quartile		64	157	60	23		
3rd quartile		78	166	73	28		

KL grade, Kellgren–Lawrence radiographic osteoarthritis grade.

### Projected cartilage area ratio and cartilage thickness

**Figure 2:**
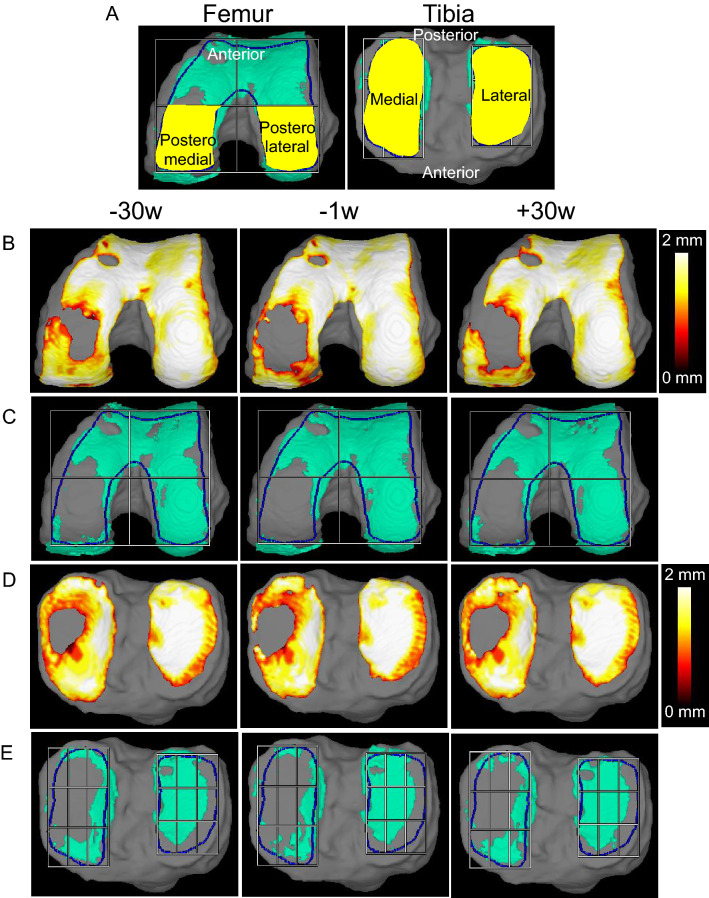
Reconstructed 3D MRI for cartilage in patient 01. (**A**) Orientation and region of interest (ROI) (yellow area). (**B**) Cartilage thickness mapping of the femur. (**C**) Projected cartilage area (thickness ≥ 1.5 mm) of the femur. (**D**) Cartilage thickness mapping of the tibia. (**E**) Projected cartilage area (thickness ≥ 1.5 mm) of the tibia.

**Figure 3:**
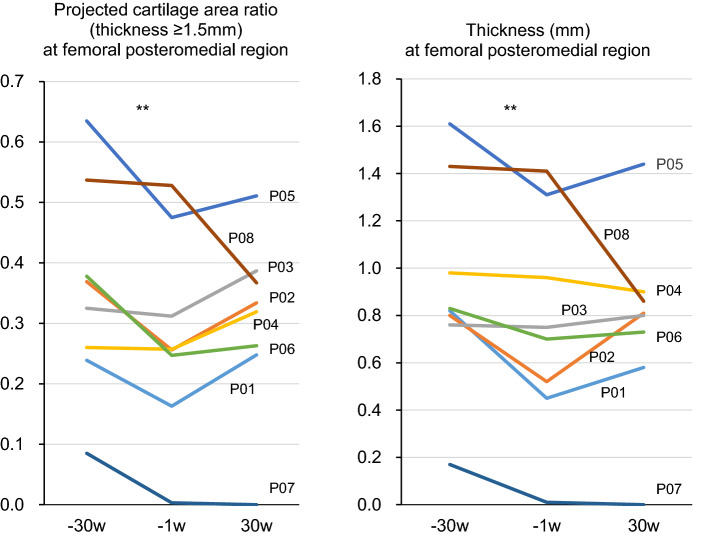
Projected cartilage area ratio (thickness ≥ 1.5 mm) and cartilage thickness at the femoral posteromedial region. The patient number is shown. **p < 0.01 between − 30 weeks and − 1 week.

The femoral posteromedial region (Fig. 2A) showed a significant difference in the alteration of the projected cartilage area ratio before and after MSC injections (Table 2). The projected cartilage area ratio significantly decreased by 0.07 at 29.8 ± 0.7 weeks prior to the MSC injections, but it did not decrease significantly in the 28.5 ± 0.5 weeks after the MSC injections (Fig. 3). The other three regions showed no significant differences in the alteration of the projected cartilage area ratio before and after the MSC injections (Table 2).

**Table 2:** Alteration in cartilage quantification before and after MSC injections.

Cartilage	Femoral				Tibial			
Region	Posteromedial		Posterolateral		Medial		Lateral	
Difference	Before^a^	After^b^	Before	After	Before	After	Before	After
**Projected cartilage area ratio (thickness ≥ 1.5 mm)**								
Patient 01	− 0.08	0.09	0.02	− 0.02	− 0.06	0.06	− 0.01	0.02
Patient 02	− 0.11	0.08	0.01	− 0.01	− 0.16	0.20	0.01	− 0.04
Patient 03	− 0.01	0.08	0.15	− 0.02	− 0.12	0.06	0.01	0.04
Patient 04	0.00	0.06	0.06	− 0.04	− 0.02	− 0.14	− 0.05	0.00
Patient 05	− 0.16	0.04	0.02	0.01	− 0.08	− 0.06	− 0.03	0.01
Patient 06	− 0.13	0.02	− 0.11	0.12	0.04	− 0.05	0.08	− 0.03
Patient 07	− 0.08	0.00	− 0.04	0.22	0.02	− 0.03	0.22	− 0.08
Patient 08	− 0.01	− 0.16	− 0.02	− 0.02	0.08	− 0.08	− 0.02	− 0.04
Median	− 0.08	0.05	0.02	− 0.02	− 0.04	− 0.04	0.00	− 0.02
1st quartile	− 0.12	0.01	− 0.03	− 0.02	− 0.10	− 0.07	− 0.03	− 0.04
3rd quartile	− 0.01	0.08	0.04	0.07	0.03	0.06	0.05	0.02
P value	0.031		0.469		0.387		0.336	
**Thickness (mm)**								
Patient 01	− 0.37	0.13	0.06	0.03	− 0.10	0.07	− 0.05	0.08
Patient 02	− 0.28	0.29	− 0.02	− 0.02	− 0.38	0.57	0.06	− 0.12
Patient 03	− 0.01	0.05	0.13	0.05	− 0.06	0.03	− 0.07	− 0.02
Patient 04	− 0.02	− 0.06	0.07	0.03	0.01	− 0.15	− 0.10	− 0.03
Patient 05	− 0.30	0.13	− 0.11	0.08	− 0.11	− 0.04	− 0.06	− 0.01
Patient 06	− 0.13	0.03	− 0.19	0.25	0.17	− 0.15	0.08	0.00
Patient 07	− 0.16	− 0.01	− 0.40	0.57	0.06	− 0.08	0.39	− 0.09
Patient 08	− 0.02	− 0.55	− 0.06	0.01	0.21	− 0.18	0.08	− 0.12
Median	− 0.15	0.04	− 0.04	0.04	− 0.03	− 0.06	0.01	− 0.03
1st quartile	− 0.29	− 0.04	− 0.15	0.02	− 0.11	− 0.15	− 0.07	− 0.11
3rd quartile	− 0.02	0.13	0.07	0.17	0.12	0.05	0.08	− 0.01
*P* value	0.098		0.148		0.422		0.184	

^a^The value at − 1 week” minus “the value at − 30 weeks.

^b^The value at 30 weeks” minus “the value at − 1 week.

The cartilage thickness showed no significant alteration before and after the MSC injections in all four regions (Table 2). The cartilage thickness at the femoral posteromedial region significantly decreased by 0.11 mm before the MSC injections, whereas it did not significantly decrease after the MSC injections (Fig. 3).

The cartilage thickness mapping of patient 1 revealed an expansion of the cartilage loss in the medial femoral condyle before the MSC injections, but cartilage was newly formed along the medial side of this cartilage loss area after the MSC injections (Fig. 2B). The projected cartilage area of the femur showed patterns similar to those of the cartilage thickness mapping (Fig. 2C). The cartilage thickness mapping for the tibia revealed an expansion of the cartilage loss area before the MSC injections, but cartilage was newly formed along the medial side of cartilage loss area after the MSC injections (Fig. 2D). The cartilage area (thickness ≥ 1.5 mm) of the medial tibia showed no obvious differences before and after the MSC injections (Fig. 2E).

### Clinical scores

**Figure 4:**
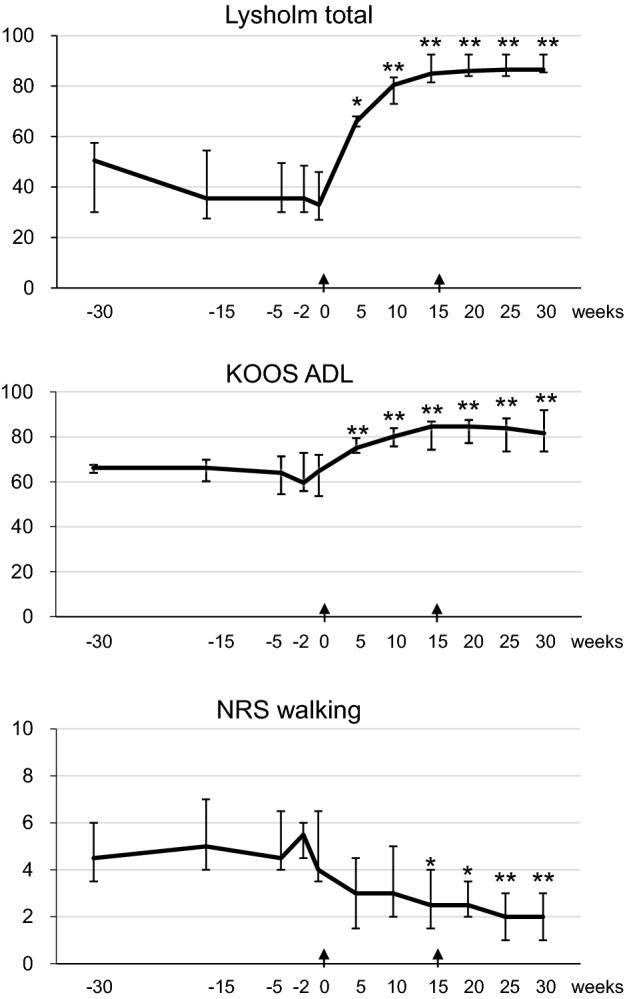
Clinical scores. Lysholm score (total) and KOOS (ADL) ranged from 0 to 100, with lower scores indicating more severe symptoms. NRS (walking) ranged from 0 to 10, with higher scores indicating more severe symptoms. MSCs were injected at time 0 and again at 15 weeks (arrow). At time 0 and 15 weeks, clinical scores were evaluated just before the MSC injections. Data are shown as median and IQR. The sample number is 8 for each outcome. *p < 0.05, **p < 0.01 with a value at time 0. Alterations in cartilage quantification before and after injections of mesenchymal stem cells into osteoarthritic knees.

The total Lysholm Knee Score increased significantly at 5 weeks and showed a further increase at 10 weeks; this latter increase was maintained at 30 weeks (Table 3, Fig. 4). The KOOS score was significantly increased at 5 weeks for symptoms, pain, and activities of daily living (ADL), and at 15 weeks for sports/recreational activities and quality of life (QOL). The KOOS for all categories was maintained at 30 weeks. The NRS score decreased significantly at 5 weeks for instability, at 15 weeks for walking, rest, and initial motion, and at 20 weeks for stairs. The NRS score for all five categories was significantly lower at 30 weeks than at time 0.

**Table 3:** Clinical scores.

	− 30w	− 15w	− 5w	− 2w	0	5w
**Lysholm**						
Total	50.5	35.5	35.5	35.5	33.0	66.0*
	(30.0, 57.5)	(27.5, 54.5)	(30.0, 49.5)	(30.0, 48.5)	(27.0, 46.0)	(64.0, 68.0)
Limping	3.0	3.0	3.0	3.0	3.0	4.0*
	(1.5, 4.0)	(0.0, 4.0)	(1.5, 4.0)	(1.5, 4.0)	(0.0, 3.0)	(3.0, 5.0)
Walker	3.5	3.5	3.5	3.5	3.5	5.0
	(2.0, 5.0)	(2.0, 5.0)	(2.0, 5.0)	(2.0, 5.0)	(2.0, 5.0)	(2.0, 5.0)
Swelling	4.0	4.0	4.0	4.0	2.0	8.0*
	(1.0, 6.0)	(1.0, 6.0)	(1.0, 6.0)	(1.0, 6.0)	(0.0, 6.0)	(6.0, 10.0)
Squatting	2.0	2.0	2.0	2.0	2.0	2.0
	(2.0, 2.0)	(2.0, 2.0)	(2.0, 2.0)	(2.0, 2.0)	(2.0, 2.0)	(2.0, 4.0)
Blockage	10.0	10.0	10.0	10.0	10.0	12.5
	(10.0, 12.5)	(10.0, 10.0)	(10.0, 10.0)	(10.0, 10.0)	(10.0, 12.5)	(10.0, 15.0)
Stairs	2.0	2.0	2.0	2.0	2.0	6.0
	(2.0, 2.0)	(2.0, 4.0)	(2.0, 4.0)	(2.0, 4.0)	(2.0, 4.0)	(4.0, 6.0)
Instability	15.0	5.0	5.0	5.0	5.0	17.5*
	(5.0, 20.0)	(2.5, 17.5)	(2.5, 17.5)	(2.5, 17.5)	(2.5, 15.0)	(12.5, 22.5)
Pain	5.0	5.0	5.0	5.0	5.0	15.0**
	(5.0, 7.5)	(2.5, 5.0)	(5.0, 5.0)	(5.0, 5.0)	(2.5, 5.0)	(10.0, 15.0)
**KOOS**						
Symptoms	57	57	57	55	57	70*
	(45, 66)	(46, 66)	(50, 59)	(50, 66)	(45, 64)	(57, 79)
Pain	58	57	49	54	50	65**
	(53, 61)	(53, 67)	(42, 60)	(38, 58)	(39, 61)	(57, 79)
ADL	66	66	64	60	65	75**
	(64, 68)	(60, 70)	(54, 71)	(56, 73)	(54, 72)	(73, 79)
Sports/recreation	25	30	38	28	28	45
	(20, 38)	(18, 48)	(23, 43)	(18, 33)	(10, 48)	(33, 50)
QOL	25	22	34	28	31	50
	(19, 41)	(19, 38)	(19, 44)	(22, 38)	(28, 44)	(28, 56)
**NRS**						
Walking	4.5	5.0	4.5	5.5	4.0	3.0
	(3.5, 6.0)	(4.0, 7.0)	(4.0, 6.5)	(4.5, 6.0)	(3.5, 6.5)	(1.5, 4.5)
Rest	2.0	3.0	2.5	3.0	2.0	1.5
	(1.0, 3.0)	(1.5, 4.0)	(1.0, 3.0)	(2.0, 3.0)	(1.5, 3.0)	(1.0, 2.5)
Initial motion	3.5	6.0*	4.5	4.0	3.5	3.0
	(3.0, 4.0)	(5.0, 7.0)	(3.0, 6.0)	(3.5, 5.0)	(3.0, 5.5)	(2.0, 3.5)
Stair	5.5	7.0	6.5	6.0	6.5	6.0
	(4.5, 6.5)	(6.0, 8.5)	(6.0, 7.5)	(4.5, 7.0)	(4.5, 8.0)	(4.0, 6.5)
Instability	6.5	8.0	7.5	7.0	7.5	6.5*
	(5.5, 8.0)	(7.0, 9.0)	(5.0, 8.0)	(6.0, 8.0)	(5.0, 9.0)	(3.5, 7.0)

	10w	15w	20w	25w	30w	
**Lysholm**						
Total	80.5**	85.0**	86.0**	86.5**	86.5**	
	(73.0, 83.5)	(81.5, 92.5	(84.0, 92.5)	(84.0, 92.5)	(85.5, 92.5)	
Limping	5.0**	5.0**	5.0**	5.0**	5.0**	
	(4.0, 5.0)	(5.0, 5.0)	(5.0, 5.0)	(5.0, 5.0)	(5.0, 5.0)	
Walker	5.0	5.0	5.0	5.0	5.0	
	(2.0, 5.0)	(3.5, 5.0)	(3.5, 5.0)	(3.5, 5.0)	(3.5, 5.0)	
Swelling	8.0**	10.0**	10.0**	10.0**	10.0**	
	(6.0, 10.0)	(6.0, 10.0)	(8.0, 10.0)	(6.0, 10.0)	(8.0, 10.0)	
Squatting	4.0*	4.0*	4.0	4.0	4.0	
	(3.0, 4.0)	(2.0, 4.0)	(4.0, 4.0)	(4.0, 4.0)	(4.0, 4.5)	
Blockage	15.0	15.0*	15.0*	15.0*	15.0*	
	(12.5, 15.0)	(15.0, 15.0)	(15.0, 15.0)	(15.0, 15.0)	(15.0, 15.0)	
Stairs	6.0	6.0*	6.0*	6.0*	6.0*	
	(4.0, 6.0)	(6.0, 6.0)	(6.0, 6.0)	(6.0, 6.0)	(6.0, 6.0)	
Instability	22.5**	25.0**	25.0**	25.0**	25.0**	
	(17.5, 25.0)	(22.5, 25.0)	(22.5, 25.0)	(25.0, 25.0)	(25.0, 25.0)	
Pain	20.0**	20.0**	20.0**	20.0**	20.0**	
	(17.5, 22.5)	(17.5, 25.0)	(17.5, 25.0)	(20.0, 25.0)	(20.0, 25.0)	
**KOOS**						
Symptoms	68	73*	71**	75**	77**	
	(59, 75)	(70, 77)	(68, 84)	(70, 88)	(71, 88)	
Pain	64	64**	75**	78**	82**	
	(51, 78)	(54, 88)	(64, 78)	(64, 86)	(65, 92)	
ADL	80**	85**	85**	84**	82**	
	(76, 84)	(74, 87)	(77, 88)	(74, 88)	(74, 92)	
Sports/recreation	45	53*	50*	53*	55*	
	(25, 55)	(40, 58)	(40, 53)	(33, 63)	(43, 66)	
QOL	41	50*	63**	50**	56**	
	(31, 59)	(31, 59)	(34, 72)	(31, 72)	(41, 72)	
**NRS**						
Walking	3.0	2.5*	2.5*	2.0**	2.0**	
	(2.0, 5.0)	(1.5, 4.0)	(2.0, 3.5)	(1.0, 3.0)	(1.0, 3.0)	
Rest	1.0	1.0*	0.0**	0.5*	0.0*	
	(0.5, 1.5)	(0.0, 2.0)	(0.0, 1.0)	(0.0, 1.0)	(0.0, 1.0)	
Initial motion	3.0	3.0*	2.5*	1.5*	2.5*	
	(1.5, 3.5)	(1.5, 3.0)	(1.0, 3.0)	(1.0, 3.5)	(0.5, 3.0)	
Stair	3.5	5.5	3.0**	3.0**	3.0**	
	(2.5, 6.0)	(2.5, 6.0)	(3.0, 4.0)	(2.5, 4.5)	(2.0, 4.5)	
Instability	6.0*	3.5**	3.0**	4.0**	3.5**	
	(3.5, 7.0)	(2.5, 6.0)	(3.0, 3.0)	(3.0, 5.5)	(2.0, 4.5)	

Data are shown as “median(1st quartile, 3rd quartile)”.

*p < 0.05, **p < 0.01 with a value at time 0 (n = 8).

### Adverse events

A total of 8 mild adverse events were recorded among the 8 patients. MSC injection could not be ruled out as the cause of two adverse events. Patient 02 reported knee pain for 1 week just after the first injection. Patient 01 reported itching in both hands that occurred 1 week after the first injection and lasted for one week. Adverse events considered not associated with MSC injection were thigh pain, nausea, vomiting, and rash before the injections and contralateral knee pain and shoulder cuff tear after the injections.

## Discussion

Synovial MSC injections significantly inhibited alterations in the projected cartilage area ratio in the femoral posteromedial region at 30 weeks after injection. Our prospective interventional study therefore achieved its predetermined primary outcome. Synovial MSC injections also significantly improved the patients’ clinical scores, with no major adverse events that required study termination.

The software we developed for 3D analysis of knee MRI has eight main functions: (1) it automatically extracts bones and cartilage; (2) it constructs bones and cartilage in three dimensions; (3) it projects the femoral and tibial cartilage in a plane; (4) it sets the ROI of four adjacent cartilage regions for the femoral cartilage and two separate cartilage regions for the tibial cartilage based on the morphology of the bones; and (5) it quantifies the projected cartilage area ratio and cartilage thickness. This is the first report in which we have used this software to analyze data from an interventional study.

The MRI in this study had an in-plane resolution of 0.31 × 0.31 mm and a partition thickness of 0.36 mm. This MRI measurement theoretically detects differences in depth and width of 0.31 mm. However, we have not been able to validate that the 0.31 mm depth and width can actually be detected using a human knee or a phantom of this shape.

Initially, the software needed manual correction for cartilage extraction due to insufficient accuracy. For neural network training, we randomly chose 10 healthy volunteers and 103 patients with knee pain, and two doctors manually segmented the data. The segmentation data were then used to train the neural network. We subsequently ran a validation test for our algorithm by randomly selecting 108 of the 113 subjects for training, while the other 5 subjects were used for a validation test by computing the Dice similarity coefficient^16^. The Dice coefficient was approximately 0.9, indicating a high knee cartilage segmentation accuracy^2^.

We had previously investigated the reproducibility of 3D MRI analysis by performing duplicate MRI scans of 10 knees, and we determined a reproducibility of 0.01–0.03, with a 95% confidence interval (CI), for the absolute value of the difference at the femoral posteromedial region the projected cartilage area ratio (thickness ≥ 1.5 mm) and a reproducibility of 0.03–0.05 mm in the cartilage thickness. The alterations in cartilage quantification exceeded these differences in many of the patient-specific values. This was a fully automatic analysis, so any inter-measurement error probably arose largely due to the MRI, and especially due to slight patient body movements. However, this study investigating the inter-scan measurement error was performed on volunteers with normal knees and has not yet been performed on patients with Kellgren–Lawrence radiographic osteoarthritis grades 2–4, which were the subjects of this study.

Our patient recruitment focused on selection of patients who were more likely to have reduced cartilage thickness based on past X-rays. At the femoral posteromedial region, the cartilage thickness had decreased by 0.15 mm in the 30 weeks before the MSC injections. If the cartilage thickness were to change at that pace for 2 years, the cumulative decrease would be 0.52 mm. A 3D MRI analysis of OA knees by Eckstein et al. indicated a decrease in cartilage thickness at the central medial femur of 0.18 mm over 2 years in knees with radiographic evidence and pain progression (n = 194)^17^. Our results showed that the cartilage thickness loss was approximately three times that value, which may reflect our selection of patients with a high probability of progressive cartilage loss based on previous X-rays and our exclusion of five patients who showed no decrease in the initial MRI analysis.

We administered 20 × 10^6^ synovial MSCs per injection. Prior to this clinical study, we conducted a clinical trial in which we transplanted synovial MSCs for repair of a degenerative meniscus tear using 20 to 60 × 10^6^ cells and a similar cell culture method to this one. The results confirmed that a single injection containing 20 × 10^6^ synovial MSCs was an effective cell dose.

We injected synovial MSCs twice at 15-week intervals. An effect of the second injection was observed in some clinical scores, but most patients showed no further improvement beyond the effects of the first injection. One possible explanation for this result is that cryopreservation reduced the potential of the cells to function as MSCs. However, our in vitro experiments indicated that human synovial MSCs cryopreserved in 95% FBS and 5% DMSO maintained their capacity for colony formation and their chondrogenic potential at the same levels observed in cells prior to cryopreservation^14^. These findings support the possibility that a second injection might not be necessary in the 30-week period.

Regarding the definition of MSCs, the International Society for Cell Therapy (ISCT) position paper states the following: (1) MSCs adhere to plastic dishes when maintained under standard culture conditions, (2) MSCs are CD105, CD73, and CD90 positive and CD45, CD34, CD14, CD11b, CD79a, CD 19, and HLA-DR negative and (3) MSCs differentiate into chondrocytes, adipocytes, and osteoblasts in vitro^18^. We prepared synovial MSCs by plating synovial nucleated cells at a relatively low density. We then selected cells that adhered to plastic dishes and further expanded them by colony formation. We did not test for multipotency, which requires 2–3 weeks, since we used primary fresh autologous synovial MSCs for the first injection. We also did not examine surface antigens because our numerous previous investigations have verified that cells that form colonies by this method have surface antigens comparable to MSCs and differentiate into chondrocytes, adipocytes, and osteoblasts in vitro^19–21^.

In one patient, a bacterial test of the HBSS used to transport the synovial tissues was suspected to be positive for contamination. However, bacterial tests of the synovial MSCs after 14 days of culture were negative for bacteria, and the behavior of these cells was similar to that of the cells from the other patients. These inconsistent bacterial test results could have two potential causes. The first is that the bacteria were not truly present, but an improper bacterial testing technique gave a false positive reading. The HBSS used to transport synovial tissues gave a negative bacterial test result for the thioglycolate (TGC) medium but a positive test result for the soybean casein digest (SCD) medium, and *Staphylococcus* species other than *Staphylococcus aureus* were detected. Staphylococci are known to grow at a high rate on TGC medium, and this suggests that the testing of the SCD medium was not performed properly. The second possibility is that the bacteria were attached to and present in the synovial tissue at the time of transport, but the antibiotics used during culture eliminated the bacteria. Since bacteria were no longer detected in the test 8 days after the cell culture, the number of bacteria, if any, was likely very small.

Two adverse events occurred that could not be ruled out as having arisen from the MSC injection. One was knee pain that lasted for 1 week after the first injection in Patient 02. This symptom was accompanied by local heat and hydrarthrosis of the knee, but it improved within 1 week. This adverse effect was considered to be a result of some factors produced by the injected MSCs that had caused a transient inflammation. The other adverse effect was itching in both hands in Patient 01. This occurred 1 week after the first injection and lasted one week. Patient 01 had known allergies to some allergens, such as crustaceans and cedar pollen. Some factors produced by the injected MSCs might therefore have induced itching in both hands.

The synovium is a thin layer of tissue that lines the joint space and covers the subsynovium. The synovium, along with the subsynovium, is commonly used to prepare synovial MSCs because of the difficulty in separating only the synovium layer from the subsynovial tissue. In OA knees, the subsynovial tissue at the suprapatellar pouch primarily consists of fibrous tissue, whereas the subsynovial tissue at the infrapatellar fat pad is primarily adipose tissue^22^. The properties of MSCs derived from these two synovial structures also differ, with adipose synovium-derived MSCs having properties intermediate between fibrous synovium-derived MSCs and subcutaneous adipose-derived MSCs^19^. Five review papers that have summarized reports of intra-articular injection of MSCs in OA knees^4,5,23–25^, have mentioned reports of IFP-derived MSCs injected into OA knees^12^, but no previous mention was made of injection of fibrous synovium-derived MSCs into OA knees. Furthermore, no previous reports have examined the effects of MSC injection using 3D MRI analysis. This study is the first report of intra-articular injection of fibrous synovium-derived MSCs into OA knees and analysis of changes in cartilage measurements by 3D MRI analysis.

Bone marrow and adipose tissue, in addition to synovium, have been reported as cell sources of MSCs for injection into OA knees (3–5). We have previously shown that the gene expression profile in synovial MSCs after monolayer culture was more similar to that in chondrocytes than that in bone marrow and adipose MSCs^26^. We also used in an in vitro differentiation model to show that the chondrogenic differentiation potential was higher in synovial and bone marrow MSCs than in adipose MSCs^21^. Similar results were also shown in an animal model following transplantation of MSCs into cartilage defects^27^. However, since the effects of MSCs injected into OA knees will be due to trophic factors produced by the MSCs in response to the OA environment^11^, the results of our previous comparative studies may not reflect the effectiveness of MSCs in the treatment of OA knees. No comparative studies have been performed that would indicate which MSCs are more effective for OA knees, and this should be clarified in the future.

The subjects in this study were older than 51 years (median age 70 years). Several reports have indicated that the functioning of MSCs, mainly derived from bone marrow, is lower when the MSCs are obtained from elderly donors^28^. We have previously compared the characteristics of synovial MSCs from donors with an average age of 20 years who underwent anterior cruciate ligament reconstruction versus MSCs from donors with an average age of 70 years who underwent total knee arthroplasty. We found no significant differences between the MSCs from the younger and older donors in terms of proliferation, colony formation, surface epitope expression, or in vitro chondrogenic differentiation potential^19^. In the current study, an age effect was observed in the MSCs from the elderly donors, but it is currently unclear whether these MSCs are less effective than MSCs from younger donors in terms of suppressing cartilage loss in patients with progressive OA.

We previously attempted to examine the behavior and function of synovial MSCs injected into the knee in a rat OA model to investigate the mechanism of MSC-treated cartilage loss^11^. Cell tracking assays showed that the majority of the injected MSCs migrated to the synovium and that the cells maintained their MSC properties without differentiating into other lineages. Species-specific gene analysis to examine the gene expression changes in human synovial MSCs that migrated to the rat synovium indicated that exogenous synovial MSCs acted as anti-inflammatory agents through TSG-6 expression^29^, as lubrication agents by PRG-4 expression^30^, and as facilitators of cartilage matrix synthesis by BMP expression^31^.

The cartilage loss before the injection was not suppressed after the injection in one of the eight knees. In this case, the effect of factors that decreased the cartilage matrix exceeded the effect of MSC injection that increased cartilage matrix. Factors commonly leading to cartilage loss include obesity, high activity, varus alignment of the knee, and medial meniscus dysfunction (meniscus injury and extrusion)^32^.

Autologous synovial MSCs from different donors differ in their proliferative^33^ and chondrogenic potential^20^ and in their quality. The use of allogeneic MSCs can overcome these limitations, but the extent of immune rejection needs to be verified when allogeneic cells are used as intra-articular injection therapy for OA knees. Alternatively, the use of MSCs derived from induced pluripotent stem cells (iPSCs) could enable quality control with batch-to-batch consistency^34^, since they are derived from the same parental iPSC lineage and possess greater proliferative capacity^35^. These iPSC-derived MSCs are expected to have a higher inhibitory effect on OA progression.

MSCs supply important paracrine factors, including exosomes, miRNAs, cytokines, and lncRNAs, as well as nutritional and mitochondrial factors^36,37^. These factors are important in the mechanisms that utilize the inflammatory environment to regulate the immune system and prevent damage to tissues in many organs^38,39^. Lian et al. reported that the paracrine profile of MSCs in a mouse allograft model of heart transplantation rejection was tightly regulated by the RAP1/NFkb signaling pathway and that the telomerase-associated RAP1 protein was impaired in aged MSCs, resulting in reduced immunosuppression^40,41^. A multifaceted and in-depth understanding of MSCs and their derivatives will be valuable for the future advancement of OA treatment.

This study had three limitations. One was the small number of patients who received MSC injections. We require more patients for the next pivotal clinical trial. The second limitation is that we did not have a control group for injection with a cell-free vehicle; therefore, the effects of intra-articular bleeding after synovial harvest, injection of 0.5 mL autologous serum, or use of the placebo^42^ cannot be neglected in our assessments. The third limitation is that the versions of software used for 3D MRI analysis were different for the screening and for the final quantification. In the one year that elapsed during the screening and analysis in this study, the software improved significantly.

## Conclusion

Fully automatic 3D MRI analysis showed that synovial MSC injections suppressed cartilage loss in the knees of patients with progressive OA.

## Data Availability

The datasets used and/or analyzed during the current study are available from the corresponding author on reasonable request.

## Abbreviations

MSC: Mesenchymal stem cell
OA: Osteoarthritis
KOOS: Knee Injury and Osteoarthritis Outcome Scale
NRS: Numerical Rating Scale
MRI: Magnetic resonance imaging
ROI: Region of interest
IFP: Infrapatellar fat pad
ROM: Range of motion
CI: Confidence interval
ADL: Activities of daily living
QOL: Quality of life
